# Microwave-Based Subsurface Characterization through a Combined Finite Element and Variable Exponent Spaces Technique

**DOI:** 10.3390/s23010167

**Published:** 2022-12-24

**Authors:** Valentina Schenone, Claudio Estatico, Gian Luigi Gragnani, Matteo Pastorino, Andrea Randazzo, Alessandro Fedeli

**Affiliations:** 1 Department of Electrical, Electronic, Telecommunications Engineering, and Naval Architecture, University of Genoa, 16145 Genoa, Italy; 2 Department of Mathematics, University of Genoa, 16146 Genoa, Italy

**Keywords:** finite element, inverse scattering, Lebesgue spaces, subsurface imaging

## Abstract

A microwave characterization technique to inspect subsurface scenarios is proposed and numerically assessed in this paper. The approach is based on a combination of finite element electromagnetic modeling and an inversion procedure in Lebesgue spaces with variable exponents. The former allows for description of the measurement system and subsurface scenario with high accuracy, while the latter exploits the adaptive definition of exponent function to achieve improved results in the regularized solution of the inverse scattering problem. The method has been assessed with numerical simulations regarding two-layered environments with both planar and non-planar air–soil interfaces. The results show the capabilities of the method of detecting buried objects in different operative conditions.

## 1. Introduction

The ability to investigate subsurface scenarios is needed for a variety of applications, including geophysical reconnaissance, archaeology, mine location and detection, environmental monitoring, and ground mapping in civil applications [1,2,3,4,5,6,7,8]. In this area, ground penetrating radars (GPRs) are one of the most widely adopted tools. However, GPR data are usually represented through B-scans, which are a time-domain representation of GPR measurements. Even though some post-processing techniques can be applied to these data, such as migration techniques [1,9], classical GPRs often require experienced users to interpret data. Moreover, they only provide a qualitative reconstruction of subsurface scenarios, without allowing for a precise dielectric characterization of the inspected targets [6].

Among the various possible approaches to inspect underground structures, microwave imaging provides the ability to characterize subsoil regions based on scattering measurements collected by antennas placed over the area of interest. These techniques can be used to non-invasively determine the presence and the approximate shape of buried objects (in the case of qualitative methods [10,11,12,13,14,15,16]) or the distribution of their dielectric properties (in the case of quantitative strategies [17,18,19,20,21,22,23,24,25]).

The scenario in which prospecting is performed makes the imaging problem particularly challenging, especially focusing on quantitative inverse scattering methods. Indeed, in addition to the general issues of the inverse scattering problem, which is inherently ill-posed and nonlinear [4], there are other issues that are typical of this configuration. First, unlike other applications where it is possible to place antennas around the entire survey area, here, antennas can only be placed on the survey line above the ground or in boreholes, reducing the available information to solve the problem [26,27]. In addition, the layering of the environment is another critical element; for instance, probes are positioned at a certain distance from the ground, and the presence of discontinuities in the dielectric properties of the scattering environment further adds a level of complexity due to the insurgence of reflection phenomena [28].

In order to reconstruct accurate images of objects in the subsurface environments, imaging techniques that integrate electromagnetic models in these settings have been proposed in the literature. Indeed, the ability to accurately model the real environment in which the diagnosis is made is crucial to obtain good results [29]. Moreover, the exploitation of a suitable model to solve the forward problem enables synthetic data to be obtained and testing methods in a simulated environment. In more detail, two main classes of mathematical models can be distinguished: analytical and numerical ones. The former are characterized by good robustness, but assuming simplified hypotheses [30,31,32,33]. The second class includes methods based on finite differences in the time domain, the finite element method, and the method of moments [34,35,36]. These classes of approaches, although more computationally expensive, are particularly advantageous when certain geometric configurations and specific soils are present in the domain. In addition, several radar-like techniques [37,38,39,40] have been recently proposed in this research area to allow soil profiling, so that retrieved soil configuration can be accurately integrated into the electromagnetic model as a priori information [41]. Finally, hybrid solutions combining analytical and numerical models have also been proposed [11].

In this paper, a quantitative imaging method based on the inversion of scattering S-parameters combined with an electromagnetic model of finite element (FE) type is proposed and applied to the diagnosis of shallow subsurface structures. In the literature, several approaches have been described to solve the nonlinear inverse scattering problem by deterministic, stochastic, and neural networks techniques [42,43,44,45,46,47]. Among the deterministic approaches, an interesting class is represented by Newton-type schemes [48,49,50,51]. In the present work, the nonlinear and ill-posed inversion problem is treated using a Newton-type technique formulated in $L^{p\left(\cdot \right)}$ Lebesgue spaces with variable exponent [52,53,54]. This is an iterative regularization procedure which is able to provide accurate diagnostic results due to an adaptive definition of the exponent function. Moreover, the FE-based electromagnetic model combined with the formulation of S-parameters [8] allows for a precise modelling of the electromagnetic problem. The approach, which was first developed for stroke imaging [54], has now been expanded to buried target detection. The main plus of the proposed approach for shallow subsurface inspection is the capability of suitably taking into account the measurements and background configuration inside the inversion procedure. Indeed, in this way, the structure of the measurement system and environments can be considered in the electromagnetic model providing valuable reconstruction results even in this challenging scenario.

The method has been validated by means of numerical data obtained with a two-layer configuration illuminated by open waveguide probes. In detail, the variation in target size, depth, and some other relevant parameters has been evaluated. Finally, a study of the method in the case of non-planar air-soil interface has been conducted; the results of inversion with a priori knowledge of the exact interface have been compared with those achieved by replacing the actual surface profile with a planar one in the embedded model.

The paper is organized as follows: in Section 2, the inverse scattering formulation along with the finite element approach and the inversion procedure are reported. The results are then presented in Section 3. Finally, the Conclusions follow in Section 4.

## 2. Formulation of the Problem

**Figure 1:**
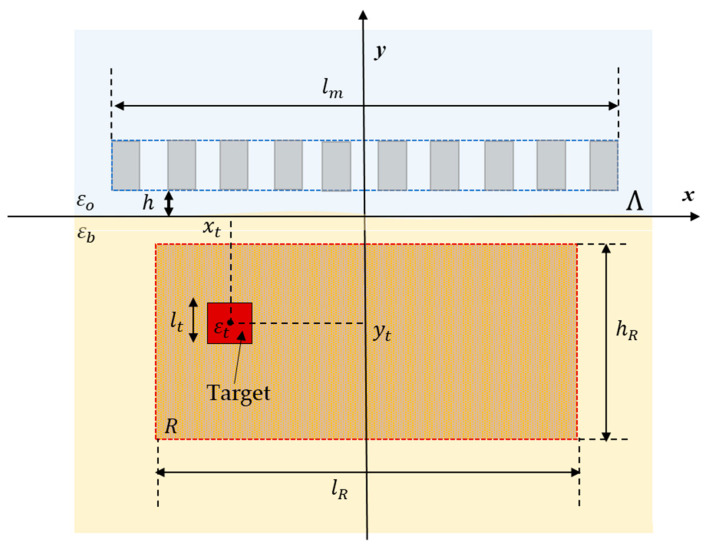
Geometry of the shallow subsurface scenario.

The shallow subsurface scenario in which detection is performed is shown in Figure 1. A multistatic and multiview system with W waveguide antennas positioned at height h from air–soil interface Λ on a measurement line of length $l_{m}$ parallel to x-axis is used to illuminate the soil. The air is modelled as vacuum (i.e., $\varepsilon_{0}≃8.85\times 10^{-12}$ F/m) and the soil is characterized by a complex dielectric permittivity $\varepsilon_{b}$.

The antennas are activated one by one, and measurements of transmission *S*-parameters are completed between the antenna ports. In addition, z-polarized fields with no components of the electric field parallel to the xy plane at an angular frequency ω are considered, and the target properties are hypothesized invariant along the z axis. Therefore, a two-dimensional environment is assumed [55].

The distribution of the dielectric permittivity of the region R containing the cross-section of the objects under test is retrieved starting from scattering S-parameters between antenna ports i and w, $\delta {\hat{S}}^{iw}=S_{tot}^{iw}-S_{inc}^{iw}$, where $S_{tot}^{iw}$ is the S-parameter measured in the presence of the target and $S_{inc}^{iw}$ is the corresponding quantity related to an empty investigation domain (assumed to be available or estimated).

Moreover, the dielectric properties of the investigation domain R can be described by the contrast function

(1) $$\tau \left(x,y\right)=\frac{\varepsilon \left(x,y\right)-\varepsilon_{b}}{\varepsilon_{b}}$$

where ε(x,y) is the complex dielectric permittivity of the configuration under test and $\varepsilon_{b}$ is the background value. In a two-dimensional setting, the scattering parameters are related to the contrast function by means of the following data equation [8]

(2) $$-\frac{j\omega \varepsilon_{b}}{2c_{i}c_{w}}\underset{R}{\iint }E_{inc}^{i}\left(x,y\right)E_{tot}^{w}\left(x,y\right)\tau \left(x,y\right)dxdy=\delta {\hat{S}}^{iw}$$

where $c_{m}$ is the incoming wave amplitude on m-th antenna port, $E_{inc}^{i}$ is the z-component of the incident electric field when the i-th antenna acts as a transmitter and $E_{tot}^{w}$ is the z-component of the total electric field when the w-th antenna transmits, i≠w. In detail, $E_{tot}^{w}$ represents the field collected in the presence of the unknown target; therefore, it is a function of the unknown contrast function itself, and this results in a nonlinear relation linking the contrast function with the scattering parameters.

Considering the measurements collected by each antenna for each view, it follows:

(3) $$\left[\begin{array}{c} -\frac{j\omega \varepsilon_{b}}{2c_{1}c_{2}}\iint_{R}E_{inc}^{1}\left(x,y\right)E_{tot}^{2}\left(x,y\right)\tau \left(x,y\right)dxdy\\ \vdots \\ -\frac{j\omega \varepsilon_{b}}{2c_{1}c_{W}}\iint_{R}E_{inc}^{1}\left(x,y\right)E_{tot}^{W}\left(x,y\right)\tau \left(x,y\right)dxdy\\ \vdots \\ -\frac{j\omega \varepsilon_{b}}{2c_{W}c_{W-1}}\iint_{R}E_{inc}^{W}\left(x,y\right)E_{tot}^{W-1}\left(x,y\right)\tau \left(x,y\right)dxdy \end{array}\right]=\left[\begin{array}{c} \delta {\hat{S}}^{12}\\ \vdots \\ \delta {\hat{S}}^{1W}\\ \vdots \\ \delta {\hat{S}}^{W\left(W-1\right)} \end{array}\right]$$

This is the nonlinear system to be inverted starting from scattering parameters measurements to retrieve the contrast function.

To handle this problem, an FE approach is applied and integrated inside the inversion performed in variable exponent Lebesgue spaces. In detail, the electromagnetic model expressed through the FE method as applied to the present inverse scattering problem is described in Section 2.1. Then, in Section 2.2, the inversion approach is presented.

### 2.1. FE Approach

A fundamental step for the development of the proposed imaging method is to define a procedure to model the electromagnetic problem, exploiting an FE formulation to compute the electric fields and S-parameters inside the inversion procedure.

**Figure 2:**
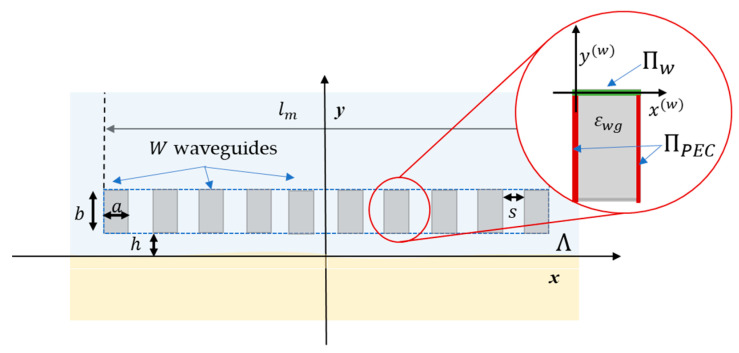
Measurement system.

In more detail, the analyzed measurement system, shown in Figure 2, is composed of a set of W open waveguides of width a and length b, filled with material of complex dielectric permittivity $\varepsilon_{wg}$. The waveguides are terminated by PEC boundaries on the lateral sides, $\Pi_{PEC}$, and a waveguide port on the top side, $\Pi_{w}.$ In each antenna, a reference system $\left(x^{\left(w\right)},y^{\left(w\right)}\right)$ is defined at the waveguide port centered at

(4) $$\left(x^{\left(w\right)},y^{\left(w\right)}\right)=\left(-\frac{l_{m}-a}{2}+\left(w-1\right)\left(s+a\right),\;h+b\right),\;\;w=1,\ldots ,W$$

where s is the mutual distance between antenna waveguides.

Assuming the i-th waveguide excited with an incoming wave of the $TE_{10}$ fundamental mode, to compute the electric field, the Helmholtz equation solution is necessary along with boundary conditions in order to take into account the exact structure of the environment and measurement system:

(5) $$\nabla_{t}^{2}E_{inc/tot}^{i}\left(x,y\right)+\omega^{2}\mu_{o}\varepsilon \left(x,y\right)E_{inc/tot}^{i}\left(x,y\right)=0$$

where non-magnetic materials are considered. The following boundary conditions must be imposed: on $\Pi_{PEC}$, Dirichlet conditions state that ${E_{inc/tot}^{i}|}_{\Pi_{PEC}}=0$; on waveguide ports, the Dirichlet and Neumann conditions are ${E_{inc/tot}^{i}|}_{\Pi_{w}}=E_{wg}^{\left(w,i\right)}$ and ${\partial E_{inc/tot}^{i}/\partial y|}_{\Pi_{w}}=\partial E_{wg,inc/tot}^{\left(w,i\right)}/\partial y^{\left(w\right)},$ with w=1,…,W where $E_{wg,inc/tot}^{\left(w,i\right)}$, i.e., the z-component of the electric field tangent to the w-th port when the i-th port is in transmitting mode, is:

(6) $$E_{wg,inc/tot}^{\left(i,w\right)}\left(x^{\left(w\right)},y^{\left(w\right)}\right)=\delta_{iw}e_{1}^{\left(i\right)}\left(x^{\left(i\right)}\right)e^{j\beta_{1}y^{\left(i\right)}}+\overset{M}{\underset{m=1}{\sum }}A_{m,inc/tot}^{\left(i,w\right)}e_{m}^{\left(w\right)}\left(x^{\left(w\right)}\right)e^{-j\beta_{m}y^{\left(w\right)}}$$

with $\delta_{iw}$ the Kronecker delta, $A_{m,inc/tot}^{\left(i,w\right)}$ the amplitude of m-th mode in wth port, $e_{m}^{\left(w\right)}$ the orthonormal modal function of $TE_{m0}$ modes in the wth waveguide, and $\beta_{m}$ the propagation constant of $TE_{m0}$ modes inside the waveguide [56].

The first term of Equation (6) represents the incoming $TE_{10}$ mode feeding the i-th port and the second one represents the outcoming $TE_{m0}$ modes inside the wth waveguide. For the solution of the forward problem, coefficients $A_{1,inc/tot}^{\left(i,w\right)}$ are left as unknowns, and the S-parameters are then retrieved as $S_{inc/tot}^{iw}=A_{1,inc/tot}^{\left(i,w\right)}$. Moreover, in the analysed configuration, absorbing boundary conditions (ABC) are required to terminate the simulation domain outside the antenna ports. To this end, the perfectly matched anisotropic absorber (PMA) has been selected [56,57].

The simulation domain includes the above-described shallow subsurface scenario comprised air and soil layers and limited by waveguide ports, waveguide PEC walls, and the PMA layer. In order to solve the forward problem with FE formulation, the domain has been partitioned in a mesh of N triangles of dimension $\Sigma_{n}$ with frontier $\Pi^{\left(n\right)}.$ Then, the electric field $E_{inc/tot}^{i}\left(x,y\right)$ in each n-th element of the mesh is formulated through first-order basis functions. It is worth highlighting that such a triangular form of subdomains gives the possibility of mapping complex structures in an accurate way, making it suitable to describe the problem at hand. In particular, the first-order triangular basis functions for each n-th triangle, $\Psi_{t}^{\left(n\right)}$, satisfy properties $\Psi_{t}^{\left(n\right)}\left(x_{u}^{\left(n\right)},y_{u}^{\left(n\right)}\right)=\delta_{ut}$ and $\sum_{t=1}^{3}\Psi_{t}^{\left(n\right)}\left(x,y\right)=1,\;\left(x,y\right)\in \Sigma_{n}$, where $\left(x_{u}^{\left(n\right)},y_{u}^{\left(n\right)}\right)$ are the coordinates of the u-th node of each n-th triangular element and are defined as follows:

(7) $$\left[\begin{array}{c} \Psi_{1}^{\left(n\right)}\left(x,y\right)\\ \Psi_{2}^{\left(n\right)}\left(x,y\right)\\ \Psi_{3}^{\left(n\right)}\left(x,y\right) \end{array}\right]={\left[\begin{array}{ccc} 1 & 1 & 1\\ x_{1}^{\left(n\right)} & x_{2}^{\left(n\right)} & x_{3}^{\left(n\right)}\\ y_{1}^{\left(n\right)} & y_{2}^{\left(n\right)} & y_{3}^{\left(n\right)} \end{array}\right]}^{-1}\left[\begin{array}{c} 1\\ x\\ y \end{array}\right],\;\;\left(x,y\right)\in \Sigma_{n}$$

The electric field in each n-th triangle can be written as:

(8) $$E_{inc/tot}^{i}\left(x,y\right)=\overset{E}{\underset{t=1}{\sum }}E_{inc/tot,t}^{i\left(n\right)}\Psi_{t}^{\left(n\right)}\left(x,y\right),\;\;\left(x,y\right)\in \Sigma_{n}$$

By considering the FE approximation of field along with the Helmholtz equation, the electric field is numerically computed following the approach in [56].

**Figure 3:**
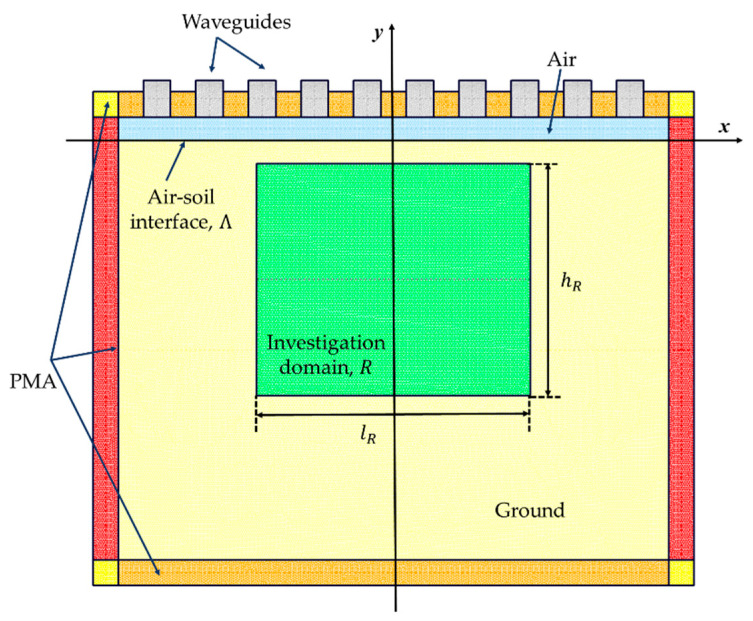
Sketch of simulation and investigation domains.

Then, in the soil region, a rectangular investigation domain R of dimension $l_{R}\times h_{R}$ centered at $\left(x_{R},y_{R}\right)$ has been defined (Figure 3), which, in its discrete representation, is a subregion of the simulation domain composed of $N_{R}$ triangles. Therefore, we can introduce the vector $\underline{\varepsilon }≅{\left[\varepsilon^{\left(1\right)},\ldots ,\varepsilon^{\left(N_{R}\right)}\right]}^{T}$, which is obtained by approximating the dielectric properties as constant inside each triangle of the domain R, thus, the corresponding contrast vector results $\underline{\tau }≅{\left[\tau^{\left(1\right)},\ldots ,\tau^{\left(N_{R}\right)}\right]}^{T}$.

In this way, starting from Equation (2), considering the FE formulation, we obtain the following system:

(9) $$\underline{D}\left(\underline{\tau }\right)≜[\begin{array}{c} -\frac{j\omega \varepsilon_{b}}{2b_{1}b_{2}}\overset{N_{R}}{\underset{n=1}{\sum }}{\underline{E}}_{inc}^{1\left(n\right)}{}^{T}[T^{\left(n\right)}]{\underline{E}}_{tot}^{2\left(n\right)}\tau^{\left(n\right)}\\ \vdots \\ -\frac{j\omega \varepsilon_{b}}{2b_{1}b_{W}}\overset{N_{R}}{\underset{n=1}{\sum }}{\underline{E}}_{inc}^{1\left(n\right)}{}^{T}[T^{\left(n\right)}]{\underline{E}}_{tot}^{W\left(n\right)}\tau^{\left(n\right)}\\ \vdots \\ -\frac{j\omega \varepsilon_{b}}{2b_{W}b_{W-1}}\overset{N_{R}}{\underset{n=1}{\sum }}{\underline{E}}_{W,inc}^{\left(n\right)}{}^{T}[T^{\left(n\right)}]{\underline{E}}_{tot}^{W\left(n\right)}\tau^{\left(n\right)} \end{array}]=\left[\begin{array}{c} \delta {\hat{S}}^{12}\\ \vdots \\ \delta {\hat{S}}^{1W}\\ \vdots \\ \delta {\hat{S}}^{W\left(W-1\right)} \end{array}\right]≜\underline{\delta \hat{S}}$$

where ${\underline{E}}_{inc/tot}^{i\left(n\right)}={\left[E_{1,inc/tot}^{i\left(n\right)},E_{2,inc/tot}^{i\left(n\right)},E_{3,inc/tot}^{i\left(n\right)}\right]}^{T}$ is the vector with the nodal values coefficients of the field in each triangle computed by means of the FE method and $\left[T^{\left(n\right)}\right]$ is a 3×3 matrix of coefficients:

(10) $$T_{ut}^{\left(n\right)}=\int_{\Sigma_{n}}\Psi_{u}^{\left(n\right)}\Psi_{t}^{\left(n\right)}dxdy,\;\;u,t=1,\ldots ,3$$

By means of Equation (8), a discrete nonlinear operator $\underline{D}\left(\underline{\tau }\right)$ linking the contrast vector with the measurements in $\underline{\delta \hat{S}}$ is defined.

### 2.2. Inversion Procedure

The imaging problem is solved by inverting Equation (9). In order to address the inversion, an inexact Newton procedure has been exploited where the space X of the unknowns is a variable exponent Lebesgue space $L^{p\left(\cdot \right)}$ (i.e., $\underline{\tau }\in X\subseteq L^{p\left(\cdot \right)}$) and the space Y of the data is a space $L^{p_{a}}$ with constant exponent (i.e., $\underline{\delta \hat{S}}\in Y\subseteq L^{p_{a}}$) [53]. In particular, since the discrete case is considered, the exponent function of unknowns’ space X turns out to be a vector $\underline{p}={\left[p^{\left(1\right)},\ldots ,p^{\left(N_{R}\right)}\right]}^{T}$, $p^{\left(n\right)}$ being the value of the exponent in each n-th triangle. The constant exponent of data space Y is set equal to its spatial average value in R, i.e., $p_{a}=\frac{1}{N_{R}}\sum_{n=1}^{N_{R}}p^{\left(n\right)}$.

**Figure 4:**
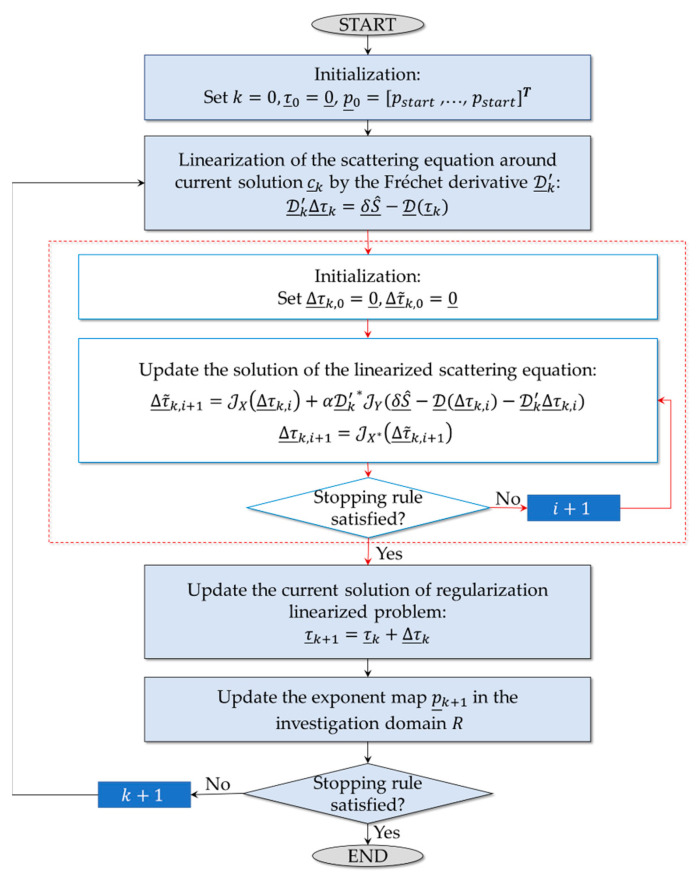
Flowchart of the inversion procedure.

The inexact Newton method solves the inverse problem in a regularized way by minimizing the following residual function:

(11) $$\Omega \left(\underline{\tau }\right)=\frac{1}{2}\|\underline{\delta \hat{S}}-\underline{D}\left(\underline{\tau }\right){\|}_{Y}^{2}$$

with $\|\cdot {\|}_{Y}$ norm in the space Y, by moving along a nonstandard gradient direction in the dual space $X^{*}$ of X. In detail, the method consists of two nested iteration cycles as summarized in the flow chart in Figure 4 [54]. At first, at each k-th iteration of the external cycle, Equation (9) is linearized around its current solution (denoted as ${\underline{\tau }}_{k}$) with null initial value ${\underline{\tau }}_{0}=\underline{0}$. Then, a Landweber-like approach in Lebesgue space $L^{p\left(\cdot \right)}$ is adopted to solve the resulting linearized problem (as shown in the red box of the flowchart). With reference to Figure 4, $J_{X}$, $J_{X^{*}}$, and $J_{Y}$ represent the duality maps of $X,\;X^{*}$, and Y, respectively. ($X^{*}=L^{p{\left(\cdot \right)}^{*}}$ is the dual space of X, with $p{\left(\cdot \right)}^{*}$ as the Hölder conjugate of p(⋅)), ${\underline{D}}_{k}^{\prime }$ is the Fréchet derivative of $\underline{D}$, and $\alpha =\|{\underline{D}}_{k}^{\prime }{\|}^{-2}$ is the step width [52,54].

Moreover, the exponent function is adaptively modified during the inversion to achieve accurate reconstruction results. In the first iteration of the external cycle, the exponent vector is set to constant values ${\underline{p}}_{0}={\left[p_{start},\ldots ,p_{start}\right]}^{T}$ since no a priori information is available. Then, at each outer iteration, the current solution ${\underline{\tau }}_{k}$ is exploited for updating $\underline{p}$, with the l-th component

(12) $$p_{k+1}^{\left(l\right)}=p_{min}+\left(p_{MAX}-p_{min}\right)\tau_{k}^{\left(l\right)}/\underset{n\epsilon N_{R}}{max}\left|\tau_{k}^{\left(n\right)}\right|,\;\;1<p_{min}\leq p_{MAX}$$

where $\left[p_{min},p_{MAX}\right]$ denotes the range of p. This way, values of p close to $p_{min}$ are assigned to the portions of R without targets leading to a reduction in ringing effects; values of p close to the maximum are in target regions where a smooth reconstruction is favored.

Finally, the two loops are stopped when the proper convergence criteria are fulfilled. In this case, the number of inner, $I_{max}$, and outer iterations, $K_{max}$, and the relative variation of the residual between two consequent steps, $\Delta_{ℛ}$, are considered as stopping rules.

## 3. Numerical Validation

In this section, numerical validation of the proposed imaging method is presented. In particular, a shallow subsurface scenario has been simulated by the FE forward solver to produce synthetic data and a reference case is described in Section 3.1. Then, the behavior of the method was investigated when some variations in the various parameters were applied. In particular, in Section 3.2 and Section 3.3, the effects and limitations on the performance of the method versus a variation in target size and target depth have been analysed, respectively. Then, in Section 3.4, the method has been studied considering different geometries and the number of targets, while in Section 3.5 the effect of the background uncertainties has been explored. Finally, in Section 3.6, some numerical tests in the presence of a non-planar air–soil interface are reported; the results achieved with exact interface surface known a priori or replaced with planar surfaces in the inversion procedure have been compared.

### 3.1. Reference Case

In numerical simulations, synthetic *S*-parameter data were provided as input to the proposed method to determine the distribution of complex dielectric permittivity within the investigation area. The FE forward solver has been exploited for the generation of numerical data.

Concerning the measurement parameters, a set of W=10 antennas was placed along the measurement line of length l=1.095 m at a distance s=55 mm from eachother and located at h=50 mm over a planar air–soil interface (Figure 1). The waveguides are filled with a material with a dielectric permittivity $\varepsilon_{\mathrm{wg}}=25\varepsilon_{0}$ and the dimensions a=60 mm in width and b=80 mm in length. The soil is characterized as dry sand with a permittivity $\varepsilon_{b}=\left(3-j10^{-3}\right)\varepsilon_{0}$.

Configurations with and without a target were simulated at frequency f=550 MHz and M=1 mode is considered. The simulation domain is discretized by Gmsh [58] using the frontal Delaunay algorithm with maximum edge length at the antenna ports and elsewhere with a size of $s_{\mathrm{wg}}=2$ mm and $s_{q}=4$ mm, respectively. In this way, total and incident *S*-parameters have been generated. Total *S*-parameters were corrupted with a multiplicative Gaussian noise of 3%.

Once synthetic data have been generated, a rectangular investigation domain R of dimension $l_{R}=60\;\mathrm{cm}$ and $h_{R}=50$ cm centered in $\left(x_{R},y_{R}\right)=\left(0,-30\right)$ cm is considered. Inside R, a coarser discretization of edges $s_{inv}=7$ mm is considered for the inversion.

The results have been evaluated using the following error metric:

(13) $$e_{Reg}=\frac{1}{N_{Reg}}\overset{N_{Reg}}{\underset{n=1}{\sum }}\frac{\left|\varepsilon^{\left(n\right)}-{\bar{\varepsilon }}^{\left(n\right)}\right|}{\left|\varepsilon_{b}\right|}$$

where Reg is the analysed region inside the investigation domain composed of $N_{Reg}$ elements, with Reg={R,tar} (i.e., whole domain R or target region tar are considered), $\varepsilon^{\left(n\right)}$ is the reconstructed dielectric permittivity in the n-th element, and ${\bar{\varepsilon }}^{\left(n\right)}$ is its reference value in the same element.

Initially, the target under consideration has a square cross-section of side $l_{t}=15\;\mathrm{cm}$, it is centered at $\left(x_{t},y_{t})=(-15,-15\right)\;\mathrm{cm}$ and characterized by a dielectric permittivity $\varepsilon_{t}=\varepsilon_{0}$. The method has been run under the following setting: $K_{max}=I_{max}=100$, $p_{start}=p_{min}$, and $p_{MAX}=2$. Furthermore, solution loops are terminated when a threshold $\Delta_{ℛ}=1\%$ is reached. Concerning the range of variation in the exponent function, a study has been conducted to find its optimum value. Specifically, $p_{min}=\left[1.2,1.9\right]$ has been considered with step 0.1.

**Figure 5:**
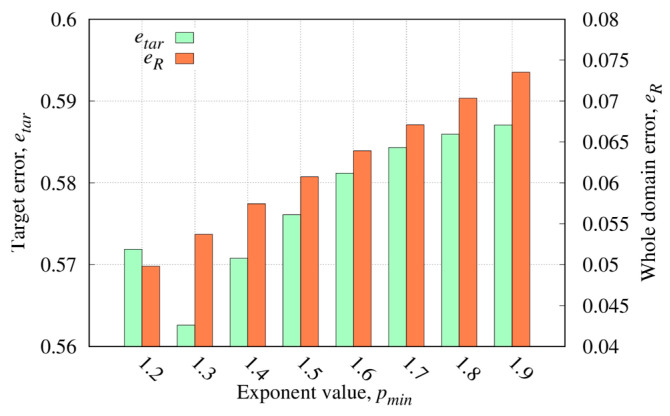
Relative reconstruction errors in all of the investigation domain R and inside the target region versus the exponent range, defined by the parameter $p_{min}$.

**Figure 6:**
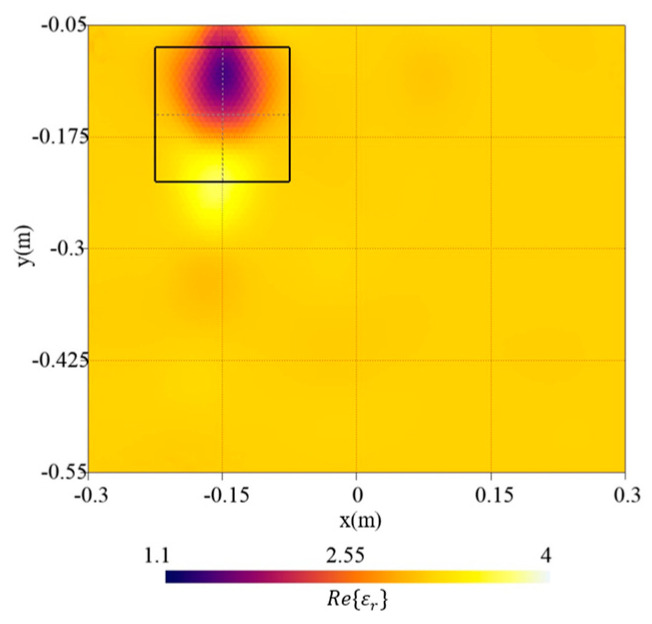
Reconstructed distribution of the real part of the dielectric permittivity, $Re\left\{\varepsilon_{r}\right\}$, in the investigation domain R together with a square box indicating the shape of the actual target. Reference case.

Figure 5 shows the errors in the whole investigation domain, $e_{R}$, and in the target region $e_{tar}$. As can be noticed, concerning the error in the investigation domain, an increment versus $p_{min}$ can be observed whereas the error inside the targets has a minimum in $p_{min}=1.3$. Therefore, $p_{min}=1.3$ has been selected since it offers the best target reconstruction and high-quality results in the investigation domain. In Figure 6, the real part of relative dielectric permittivity is reported along with the actual target shape. As can be noticed, the localization of the target is adequate, and its dielectric permittivity has been reconstructed quite well.

### 3.2. Variation in Target Size

**Figure 7:**
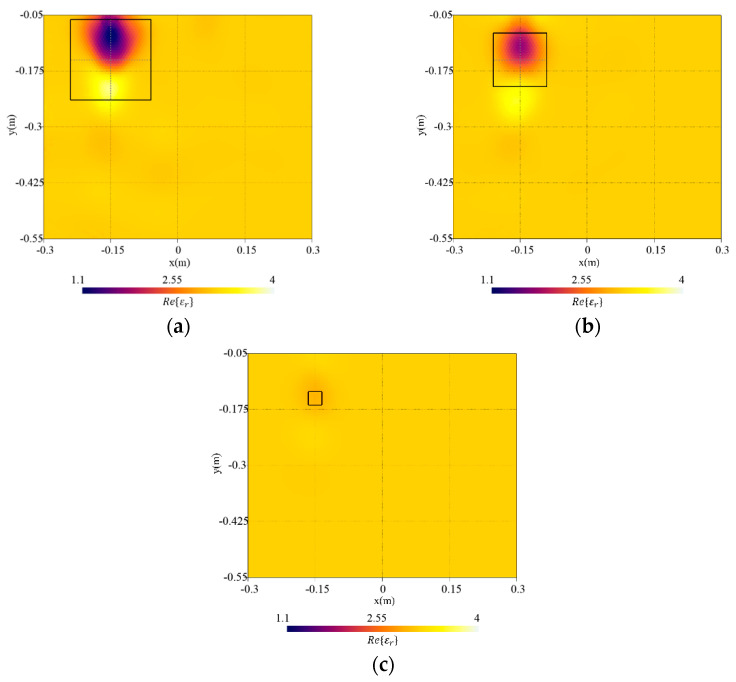
Reconstructed distribution of the real part of the dielectric permittivity, $Re\left\{\varepsilon_{r}\right\}$, in the investigation domain R together with a square box indicating the shape of the actual target. Target with side length (**a**) $l_{t}=18$ cm, (**b**) $l_{t}=12$ cm, and (**c**) $l_{t}=3$ cm.

**Figure 8:**
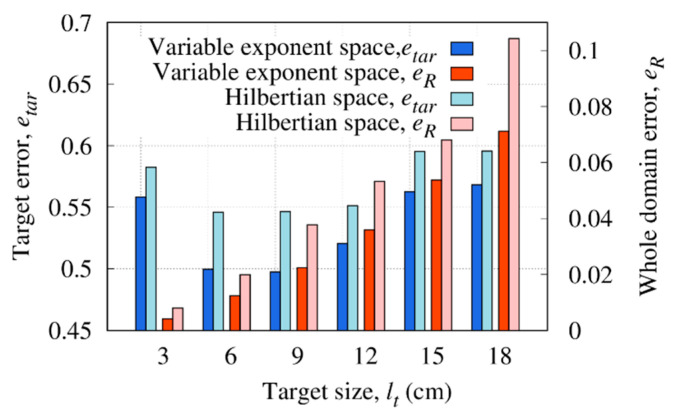
Reconstruction errors in the whole investigation domain and inside the target region versus target size, $l_{t}$ with the variable exponent approach and classical Hilbertian space.

The size of the target has been changed in the first set of numerical simulations. Specifically, the values $l_{t}\in \left[3,18\right]$ cm with a step of 3 cm have been considered for the side length (i.e., $l_{t}$ has been varied between $\left[0.095\lambda_{b},0.572\lambda_{b}\right]$ with $\lambda_{b}$ the wavelength in the soil). Figure 7 shows the reconstructed distributions of the real part of the dielectric permittivity for $l_{t}=\left\{3,12,18\right\}$ cm. In configurations with $l_{t}=18$ cm and $l_{t}=12$ cm [Figure 7a,b], the reconstruction of the dielectric permittivity is close to the actual value. A good reconstruction is obtained, and the dielectric target is clearly visible and adequately localized. Conversely, considering a smaller target with $l_{t}=3$ cm, the object is barely visible in the reconstruction [Figure 7c]. In Figure 8, the reconstruction errors computed in the investigation domain and inside the target region are reported. As can be noticed, the whole domain error $e_{R}$ tends to increase with the target size. Instead, the error on the object $e_{tar}$ has a parabolic-like behavior with a minimum at $l_{t}=9$ cm ($0.286\lambda_{b}$). In particular, very small targets are weak scatterers and become difficult to detect. Indeed, an underestimation of the dielectric properties can be observed by reducing the target’s size until the method is not able to reconstruct the object. On the contrary, slight degradation of the background reconstruction can be seen by increasing the size of the target [Figure 7a].

In addition, the method was compared with the inversion formulated in the classical Hilbertian space (i.e., $X\subseteq L^{2},\;Y\subseteq L^{2}$). By comparing the errors achieved with the proposed variable exponent space method, it can be observed that the latter approach allows an accuracy improvement both in the investigation domain and in the target under test. This proves that the inversion performed in variable exponent Lebesgue spaces leads to more accurate reconstructions. Indeed, by defining a proper map of the exponent [Equation (12)], low values are assumed in the background and values close to $p_{MAX}$ are in the region where the inhomogeneities are localized. In this way, low values better control sparsity in the background whilst a better estimation of smoothness of the object is reached by assigning relatively high values of exponent in that region.

### 3.3. Variation in Target Depth

**Figure 9:**
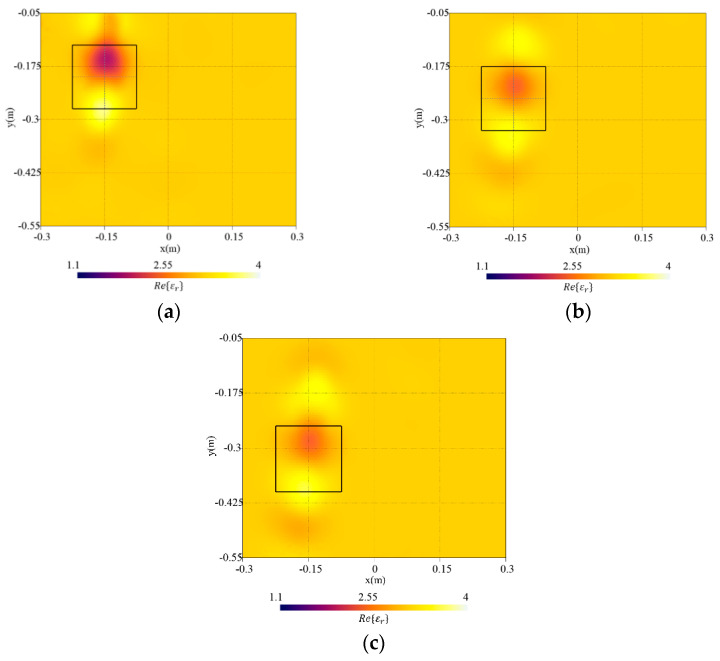
Reconstructed distribution of the real part of the reconstructed relative dielectric permittivity, $Re\left\{\varepsilon_{r}\right\}$, in the investigation domain R together with a square box indicating the shape of the actual target. Target centered at (**a**) $y_{t}=-20\;\mathrm{cm}$, (**b**) $y_{t}=-25\;\mathrm{cm}$, and (**c**) $y_{t}=-32.5\;\mathrm{cm}$.

**Figure 10:**
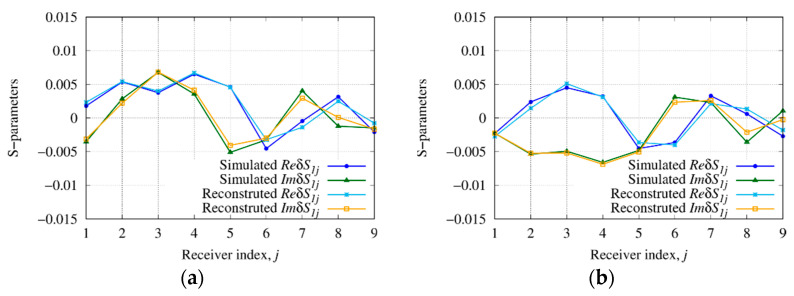
Simulated and reconstructed scattering *S*-parameters (real and imaginary part) when the first antenna acts as the source. Target centered at (**a**) $y_{t}=-20\;\mathrm{cm}$ and (**b**) $y_{t}=-25\;\mathrm{cm}$.

As a further analysis, the depth of the buried target has been varied, i.e., $y_{t}\in \left[-15,-35\right]$ cm with a step of 2.5 cm (i.e., $y_{t}\in \left[-0.477\lambda_{b},-1.112\lambda_{b}\right]$) has been considered in order to assess the effectiveness and limitations of the method depending on the depth. The reconstructed distributions of the real part of the relative dielectric permittivity, $Re\left\{\varepsilon_{r}\right\}$, are shown in Figure 9 for the cases $y_{t}=-32.5$ cm, $y_{t}=-25$ cm, and $y_{t}=-20$ cm. Moreover, in Figure 10, the scattering *S*-parameters simulated when the first antenna acts as the source are compared with those computed inside the inversion procedure from the reconstructed dielectric permittivity for cases $y_{t}=-25$ cm and $y_{t}=-20$ cm. As can be observed, the reconstructed data match with the simulated ones.

By comparing the results, as the depth increases [Figure 6 and Figure 9a,b], the target reconstruction shows a decrease in the accuracy of the dielectric properties up to the point where it is difficult to locate the object [Figure 9c].

**Figure 11:**
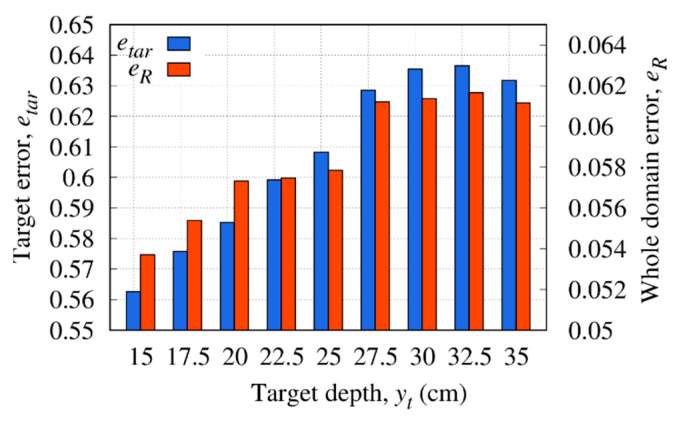
Reconstruction errors in the whole domain and inside the target versus the target size, $y_{t}$.

The reconstruction errors in the whole investigation domain and target region are shown in Figure 11. For low values of depth, the best target and whole domain error are achieved; then, by deepening the buried target, both the error in the target region and in the whole investigation domain has an upward trend. In more detail, beyond the depth of $y_{t}=-25$ cm ($y_{t}=-0.794\lambda_{b}$), the detection of the object is more difficult, and the quality of the reconstruction deteriorates as evidenced by the error trend, which for $y_{t}<-25$ cm shows $e_{R}>0.6$.

### 3.4. Effect of Different Geometries and a Different Number of Targets

In this section, the behavior of the method has been investigated considering different geometries and a different number of targets.

To this end, the target with a square-cross section of side $l_{t}=12$ cm analysed in the previous section has been considered and a second target with a circular-cross section has been introduced in the investigation domain. In detail, the second target is “vacuum-filled”, has a radius r=6 cm, and is located at $\left(x_{t2},y_{t2})=(17,-20\right)\;\mathrm{cm}$.

**Figure 12:**
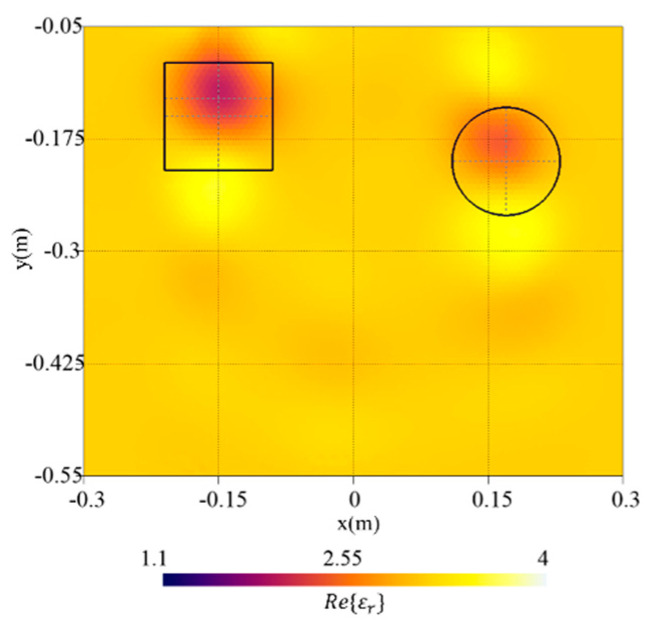
Reconstructed distribution of the real part of the dielectric permittivity, $Re\left\{\varepsilon_{r}\right\}$, in the investigation domain R together with a square box indicating the shape of the actual target. The case of two buried targets with different geometry.

Figure 12 shows the reconstructed distribution of the real part of the dielectric permittivity together with the actual shapes of the targets. As can be noticed, both targets with different geometries are correctly localized, and although in the case of the object with a circular-cross section a slight underestimation can be observed, a quite good reconstruction is achieved. Moreover, by comparing the reconstruction of the square-cross section target alone [Figure 7b] and in the presence of a second object, a slight deterioration can be observed, due to the interaction between the objects. This is confirmed by the computed reconstruction errors in the target region and in the whole domain (i.e., $e_{tar}=0.553$ and $e_{R}=0.074$), which are higher than in the single-target case (i.e., $e_{tar}=0.521$ and $e_{R}=0.035$).

### 3.5. Effect of Uncertainties in Soil Dielectric Properties

**Figure 13:**
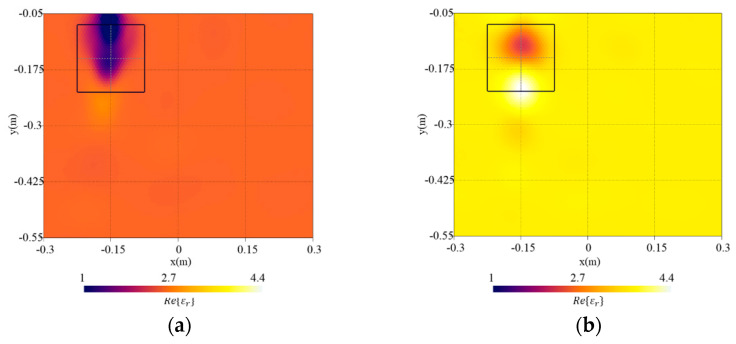
Reconstructed distribution of the real part of the dielectric permittivity, $Re\left\{\varepsilon_{r}\right\}$, in the investigation domain R together with a square box indicating the shape of the actual target. Background dielectric permittivity in the inversion procedure: (**a**) underestimation ($\varepsilon_{r,b}^{*}=\varepsilon_{r,b}-0.5$) and (**b**) overestimation ($\varepsilon_{r,b}^{*}=\varepsilon_{r,b}+0.5$).

In order to assess the robustness of the method versus an inexact knowledge of the dielectric properties of the soil, the background dielectric permittivity used inside the inversion procedure has been set to $\varepsilon_{r,b}^{*}=\varepsilon_{r,b}\pm 0.5$. All the other parameters are the same as those described in Section 3.1. In the first case, an underestimation of the background dielectric permittivity is considered whereas in the second case an overestimation is assumed. Figure 13 shows the reconstructed distributions of the real part of the dielectric permittivity. The reconstruction errors in the whole investigation domain and in the target region are shown in Table 1 (for the sake of comparison, we also reported the errors when the exact background properties are known in the inversion analysed in Section 3.1). The error in the whole investigation domain increases when an inaccurate background permittivity is considered. Moreover, a decrement of the target error can be noticed in the first case because the initial estimation of background permittivity is closer to the properties of the object.

### 3.6. Variation in Air–Soil Interface Roughness

A further study has been conducted to inspect the robustness of the method in the presence of a non-planar air–soil interface. Moreover, the results have been compared when:

- the model endowed in the inversion takes into account the exact interface profile (i.e., assuming that the surface is known a priori);
- a planar interface is considered instead (i.e., no a priori information is available).

**Figure 14:**
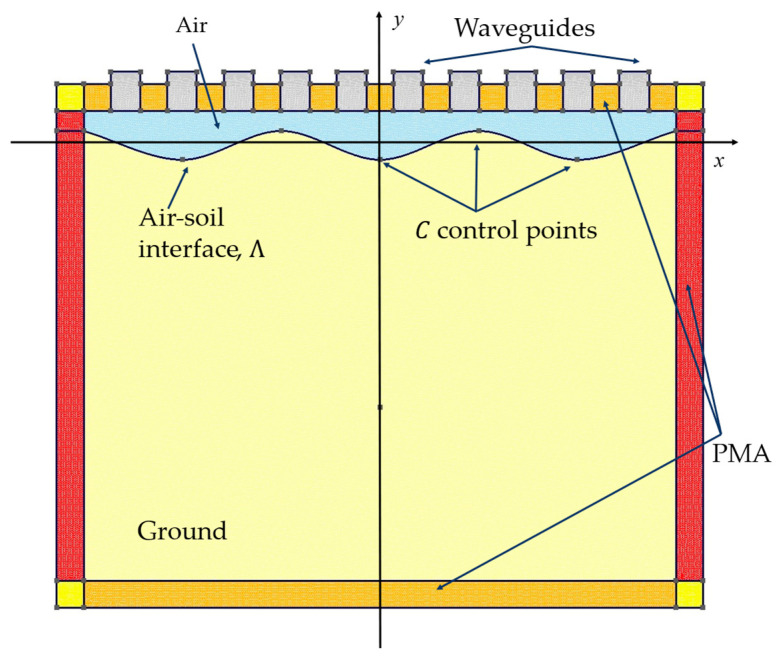
Measurement and subsurface model in the forward solver.

In the analysed measurement setting, the antennas are located at h=7 cm over the average soil level. To simulate a non-planar interface, in this set of tests, the profile of Λ is modelled with Catmull–Rom splines [59]. In particular, Λ is based on C=7 equally spaced control points located to have a no-planar air-soil interface as shown in Figure 14. The root mean square height of the interface has been varied between hrms=λ10 and hrms=λ30.

A “vacuum-filled” target (i.e., characterized by a permittivity $\varepsilon_{t}=\varepsilon_{0}$) centered at $\left(x_{t},y_{t}\right)=(-20,-20)\;\mathrm{cm}$ with $l_{t}=12$ cm has been investigated. The investigation domain is centered at $\left(x_{R},y_{R}\right)=\left(0,-32\right)$ cm. All the other parameters of the forward and inverse procedure are the same as those in the previous sections.

**Figure 15:**
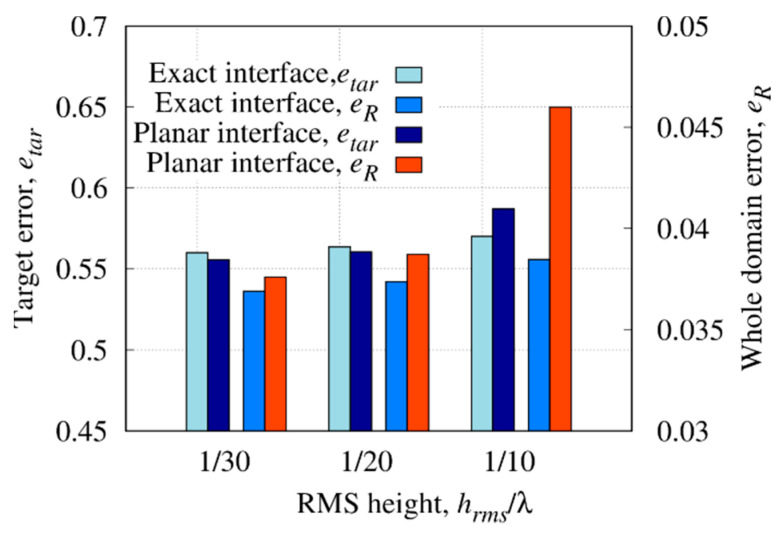
Reconstruction errors in the whole domain and inside the target versus the RMS height of the air–soil interface, $h_{rms}$.

**Figure 16:**
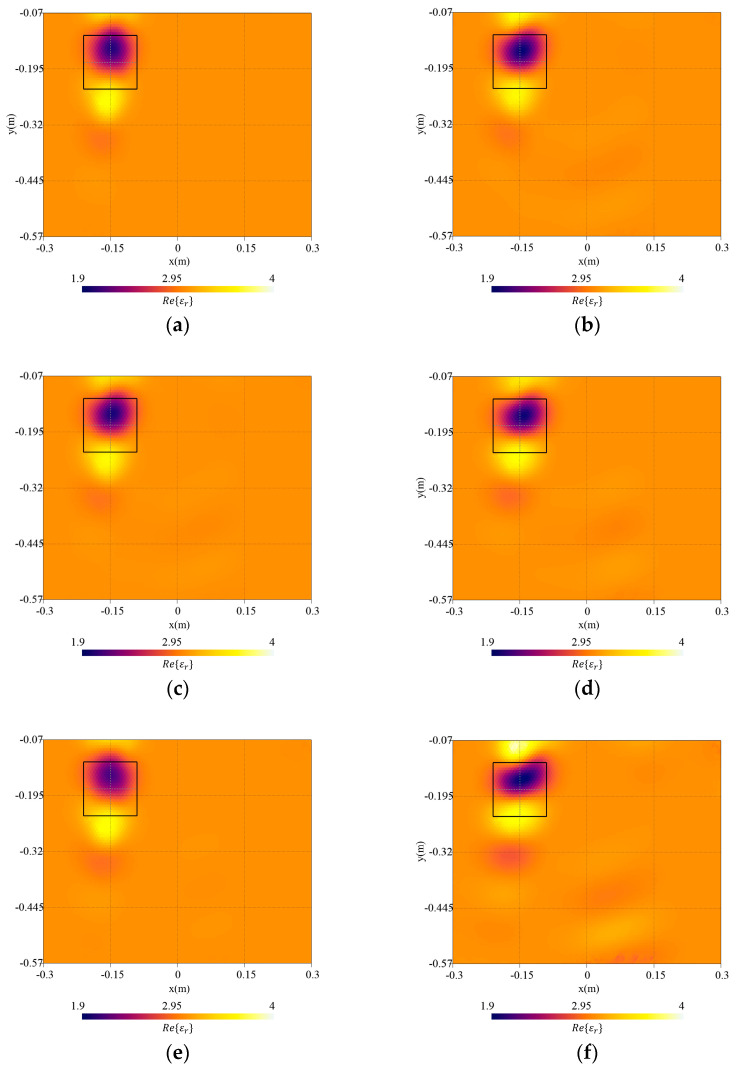
Reconstructed distribution of the real part of the complex dielectric permittivity, $Re\left\{\varepsilon_{r}\right\}$, in the investigation domain together with a square box indicating the shape of the actual target. Cases of surface with hrms=λ30 and inversion with (**a**) exact interfaces and (**b**) planar interfaces; hrms=λ20 and inversion with (**c**) exact interfaces and (**d**) planar interfaces; and hrms=λ10 inversion with (**e**) exact interfaces and (**f**) planar interfaces.

In Figure 15, the reconstruction errors in the target region and in whole investigation domain are reported. Figure 16a–c shows the real part of the reconstructed dielectric permittivity when the surface profile is considered as a priori information in the inversion and Figure 16d–f reports the reconstruction when planar interfaces are adopted in the FE-model inside the inversion procedure.

As can be noticed from the reconstruction of the real part of the permittivity, although the reconstruction is quite successful in both cases, the artifacts in the background appear more pronounced for greater interface roughness when no a priori information about the interface is assumed in the inversion. This is confirmed by the trend of the errors; the error on the object is comparable in the two cases for hrms≤λ20, while with hrms=λ10, the knowledge of the exact interface brings a significant improvement in the reconstruction; the error on the whole domain is better in all cases with a valuable improvement as the roughness increases.

This proves the potentiality of the model embedded in the inversion procedure combined with *S*-parameters formulation in various operating conditions. Indeed, an accurate structural description of the involved environments can be integrated and taken into account in the inversion procedure, leading to an enhancement in reconstruction results.

## 4. Conclusions

In this paper, a microwave imaging technique for shallow subsurface prospecting is proposed, whose aim is to provide the distribution of dielectric permittivity from scattering *S*-parameters measurements. The method combines an FE approach with an inversion procedure in Lebesgue spaces with variable exponent. In this way, the structure of the measurement system together with the environment can be accurately considered together in the electromagnetic model by the FE formulation that is incorporated into the inversion procedure. Furthermore, the adopted inversion technique, which operates in non-Hilbertian Lebesgue spaces, exploits the adaptive update of the exponent function during the inversion procedure leading to good results even in this challenging scenario.

At first, the method is tested by considering a set of waveguide probes to illuminate a “vacuum-filled” target buried in a two-layered configuration with a planar interface. The behavior and limitations of the method such as the size and depth of the target change have been studied. Moreover, the effects of geometries and the number of targets as well as the uncertainties in soil dielectric permittivity have been investigated. In addition, the behavior of the method in the case of the non-planar air–soil interface has been analyzed, as well as the effect of the a priori knowledge of the interface profile inside the inversion procedure. The potentialities of this method for shallow subsurface prospection are shown by the achieved results. Future developments include both the validation with experimentally measured data and the extension to three-dimensional settings.

## Figures and Tables

**Table 1:** Reconstruction errors in the whole domain and inside the target versus estimation of background dielectric permittivity.

Background	$\mathrm{Whole}\;\mathrm{Domain}\;\mathrm{Error},\;e_{R}$	$\mathrm{Target}\;\mathrm{Error},\;e_{tar}$
Exact estimation	0.054	0.563
Underestimation	0.187	0.378
Overestimation	0.215	0.766

## Data Availability

The numerical data presented in this study are available from the corresponding authors on request.
